# Optimization and scale up of production of the PSMA imaging agent [^18^F]AlF-P16-093 on a custom automated radiosynthesis platform

**DOI:** 10.1186/s41181-024-00247-1

**Published:** 2024-02-23

**Authors:** David Alexoff, Seok Rye Choi, Karl Ploessl, Dohyun Kim, Ruiyue Zhao, Lin Zhu, Hank Kung

**Affiliations:** 1 Five Eleven Pharma Inc., Philadelphia, PA 19104 USA; 2 Isotope Research and Production Department, Brookhaven National Laboratory, Upton, NY 11973 USA; 3 Department of Nuclear Medicine, The First Affiliated Hospital of Guangzhou Medical University, Guangzhou, 510120 Guangdong China; 4 College of Chemistry, Key Laboratory of Radiopharmaceuticals, Ministry of Education, Beijing Normal University, Beijing, 100875 China; 5 Department of Radiology, University of Pennsylvania, Philadelphia, PA 19104 USA

**Keywords:** [^18^F]AlF-P16-093, Automated synthesis, [^68^Ga]P16-093, PSMA, [^18^F]AlF^2+^

## Abstract

**Background:**

Recent advancements in positron emission tomograph (PET) using prostate specific membrane antigen (PSMA)-targeted radiopharmaceuticals have changed the standard of care for prostate cancer patients by providing more accurate information during staging of primary and recurrent disease. [^68^Ga]Ga-P16-093 is a new PSMA-PET radiopharmaceutical that demonstrated superior imaging performance in recent head-to-head studies with [^68^Ga]Ga-PSMA-11. To improve the availability of this new PSMA PET imaging agent, [^18^F]AlF-P16-093 was developed. The ^18^F-analog [^18^F]AlF-P16-093 has been synthesized manually at low activity levels using [^18^F]AlF^2+^ and validated in pre-clinical models. This work reports the optimization of the production of > 15 GBq of [^18^F]AlF-P16-093 using a custom automated synthesis platform.

**Results:**

The sensitivity of the radiochemical yield of [^18^F]AlF-P16-093 to reaction parameters of time, temperature and reagent amounts was investigated using a custom automated system. The automated system is a low-cost, cassette-based system designed for 1-pot syntheses with flow-controlled solid phase extraction (SPE) workup and is based on the Raspberry Pi Zero 2 microcomputer/Python3 ecosystem. The optimized none-decay-corrected yield was 52 ± 4% (N = 3; 17.5 ± 2.2 GBq) with a molar activity of 109 ± 14 GBq/µmole and a radiochemical purity of 98.6 ± 0.6%. Run time was 30 min. A two-step sequence was used: SPE-purified [^18^F]F^−^ was reacted with 80 nmoles of freeze-dried AlCl_3_·6H_2_O at 65 °C for 5 min followed by reaction with 160 nmoles of P16-093 ligand at 40 °C for 4 min in a 1:1 mixture of ethanol:0.5 M pH 4.5 NaOAc buffer. The mixture was purified by SPE (> 97% recovery). The final product formulation (5 mM pH 7 phosphate buffer with saline) exhibited a rate of decline in radiochemical purity of ~ 1.4%/h which was slowed to ~ 0.4%/h when stored at 4 °C.

**Conclusion:**

The optimized method using a custom automated system enabled the efficient (> 50% none-decay-corrected yield) production of [^18^F]AlF-P16-093 with high radiochemical purity (> 95%). The method and automation system are simple and robust, facilitating further clinical studies with [^18^F]AlF-P16-093.

## Introduction

Positron emission tomography (PET) using prostate-specific membrane antigen (PSMA)-targeted radiopharmaceuticals is transforming clinical care for prostate cancer (PCa) patients by providing physicians with more accurate information during staging of primary and recurrent disease (Fendler et al. 2023; Houshmand et al. 2023). The first PSMA-PET radiopharmaceuticals approved in the United States were Ga68-PSMA-11 and Pylarify®—F18 DCFPyl, followed by kit formulations of PSMA-11 (Illuccix®—Ga 68 gozetotide; Locametz®—Ga 68 gozetotide) (Carlucci et al. 2021; Voter et al. 2022; FDA 2022a, b). These PSMA radiotracers have shown similar clinical utility in both head-to-head studies (Dietlein et al. 2017; Dietlein et al. 2015) and retrospective analyses (Evangelista et al. 2022), including a recent pilot study that concluded that both [^68^Ga]Ga-PSMA-11 and [^18^F]FPyl may be suitable for selecting patients for radioligand therapy (RLT) using Pluvitco® (lutetium Lu 177 vipivotide tetraxetan, also known as [^177^Lu]Lu-PSMA-617; Fallah et al. 2023) using the same thresholding criteria (Heilinger et al. 2023).

In these studies, however, differences in image contrast between ^68^Ga- and ^18^F-PSMA-PET tracers were reported. For example, Heilinger et al. (2023) observed a twofold increase in the median contrast to noise ratio of [^18^F]DCFPyl over [^68^Ga]Ga-PSMA-11 (Heilinger et al. 2023). The authors surmised that improved image contrast was due in part to the later imaging (120 min after injection) which was possible because of the longer half-life of fluorine-18 compared to gallium-68 (109.6 min vs. 68 min) and higher injected activity (Dietlein et al. 2015; Heilinger et al. 2023). In general, higher resolution images are obtained with fluorine-18 vs gallium-68 due to the differences in their physical decay properties, the former having a higher branching fraction and lower average positron energy (Carter et al. 2020).

Given these advantages of ^18^F-PSMA, combined with the existence of a cyclotron-based supply chain providing high-activity batches of fluoride-18 throughout much of the developed world, fluoride-18 would be the preferred radionuclide for the development of any new PSMA-PET imaging agents. Radiotracers based on fluoride-18 can be produced in a centralized pharmacy and distributed widely, each batch supporting many more patients than possible with gallium-68, which is often limited to 2–4 patients/generator. Though cyclotron-produced gallium-68 is also available (Thisgaard et al. 2021), it is not yet widely available and the shorter half-life of gallium-68 limits distribution of the final product. Hence, the latest PSMA-PET agent to receive regulatory approval by the FDA in 2023, Posluma® (rh PSMA 7.3), utilizes fluoride-18 as the radionuclide for the imaging molecule in a radiohybrid design (Jani et al. 2023; Surasi et al. 2023). PSMA-1007, also an ^18^F-based tracer is in widespread clinical development though not commercially available. Its pharmacokinetics was designed to minimize background/spillover from bladder activity, a potential advantage in detection of pelvic lesions in recurrent PCa disease (Wurzer et al. 2020; Eiber et al. 2020).

[^68^ Ga]Ga-P16-093 is a second-generation PSMA-PET agent in clinical trial (Lee et al. 2022; Green et al. 2020; Bahler 2023). It has demonstrated an altered excretion pathway in humans leading to significantly lower bladder activity (Green et al. 2020; Wang 2023) as well as improved tumor detection characteristics compared to [^68^Ga]Ga-PSMA-11 in the same patient, particularly in individuals with diagnosis of a low/intermediate risk PCa (Wang 2023). Given the aforementioned advantages of fluoride-18, a ^18^F-based tracer would be preferred, and [^18^F]AlF-P16-093 was described recently (Zha et al. 2021). [^18^F]AlF-P16-093 was synthesized manually in high yield using an adaptation of the method first described by McBride et al. (2009) based on the chemical species [^18^F]AlF^2+^. [^18^F]AlF-P16-093 exhibited excellent specific binding profiles in cell, tissue and mouse tumor systems and was considered a good candidate for evaluation in humans (Zha et al. 2021).

**Fig. 1:**
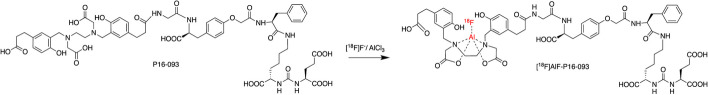
Reaction scheme with chemical structures of P16-093 and [^18^F]AlF-P16-093

Before a novel PET-agent can be investigated routinely in a clinical setting, large-scale synthesis must be demonstrated, preferably using an automated platform. [^18^F]AlF-P16-093 incorporates the acyclic chelator HBED-CC (see Fig. 1), like PSMA-11, which forms the basis for application of the [^18^F]AlF^2+^ method. Several groups have demonstrated successful large-scale, automated production of [^18^F]AlF-PSMA-11 using [^18^F]AlF^2+^ in moderate yield (< 25% not decay corrected), high radiochemical purity, and good stability in high activity batches (> 10 GBq) (Kersemans et al. 2018; Giglio et al. 2018). The goal of this work was to scale up and automate the production of [^18^F]AlF-P16-093, guided in part by these previous successes with [^18^F]AlF-PSMA-11. We report here a procedure for the large-scale production of [^18^F]AlF-P16-093 in high yield (> 50% not decay corrected) using a custom automated synthesis platform.

## Methods

### General

Several important chemicals were obtained commercially; AlCl_3_ hexahydrate (ReagentPlus®), sodium acetate trihydrate (BioUltra grade) and water (ACS reagent, for ultratrace analysis) were purchased from Sigma-Aldrich (Milwaukee, WI). Acetic acid (Certified ACS) was bought from Thermo Fisher Scientific (Fair Lawn, NJ). TLC Silica gel 60 F254 plates were obtained from Merck KGaA, Germany. Sep-Pak© Plus Light QMA (Cl-), Sep-Pak© Plus Light QMA and Oasis® HLB 6 cc/150 mg cartridges were purchased from Waters (Milford, MA). High-performance liquid chromatography (HPLC) analysis was performed on an Agilent 1100 series (Agilent, USA) equipped with NaI (TI) scintillation detector (Model 106, Ecker-Ziegler, Germany) with a reversed-phase column (Luna, C18, 150 × 4.6 mm, 5 micron, Phenomenex, USA). Lyophilization was carried out using a SP VirTis Genesis Pilot Freeze Dryer (Warminster, PA). F18 Activity was measured using a Packard Cobra II auto gamma counter (Perkin Elmer, Waltham, MA) or a Capintec (Floral Park, NJ) Model CRC 127-R dose calibrator. Free ligand, P16-093, was synthesized as described previously (Zha et al. 2018).

[^18^F]fluoride was produced with the IBA cyclotron, Cyclone 18/9 (Louvain-La-Neuve, Belgium), via the ^18^O (p, n) ^18^F reaction at the University of Pennsylvania with a niobium target and PEEK delivery lines. For “^18^F-rinse” studies, [^18^F]fluoride was obtained by rinsing the target and delivery lines with 1 mL of [^18^O]O-water after a previous production run. [^18^O]O-water (> 97%) was purchased from Huayi Isotopes (Changshu, China) or ABX (Radeberg, Germany).

Stopcock manifolds (polypropylene, PT-188, Huayi Isotopes) for automated synthesis were purchased from Nucmedcor (San Francisco, CA). Flexelene® and silicone (Pharma 50) tubing for synthesis cassettes were purchased from Eldon James (Fort Collins, CO) and Cole-Parmer (Vernon Hills, IL) respectively. The reaction vessel for automated synthesis was made of a Wheaton® (Millville, NJ) 10 mL E-Z EX-TRACTION™ vial, a threaded (22–400) phenolic cap (DWK, Millville, NJ) and a silicone septum with PTFE lining (20 × 3 mm) purchased from Tisch Scientific (Cleveland, OH).

Statistical data comparison was done using a Student’s T-Test (Microsoft Excel).

### Custom automated radiosynthesis platform

**Fig. 2:**
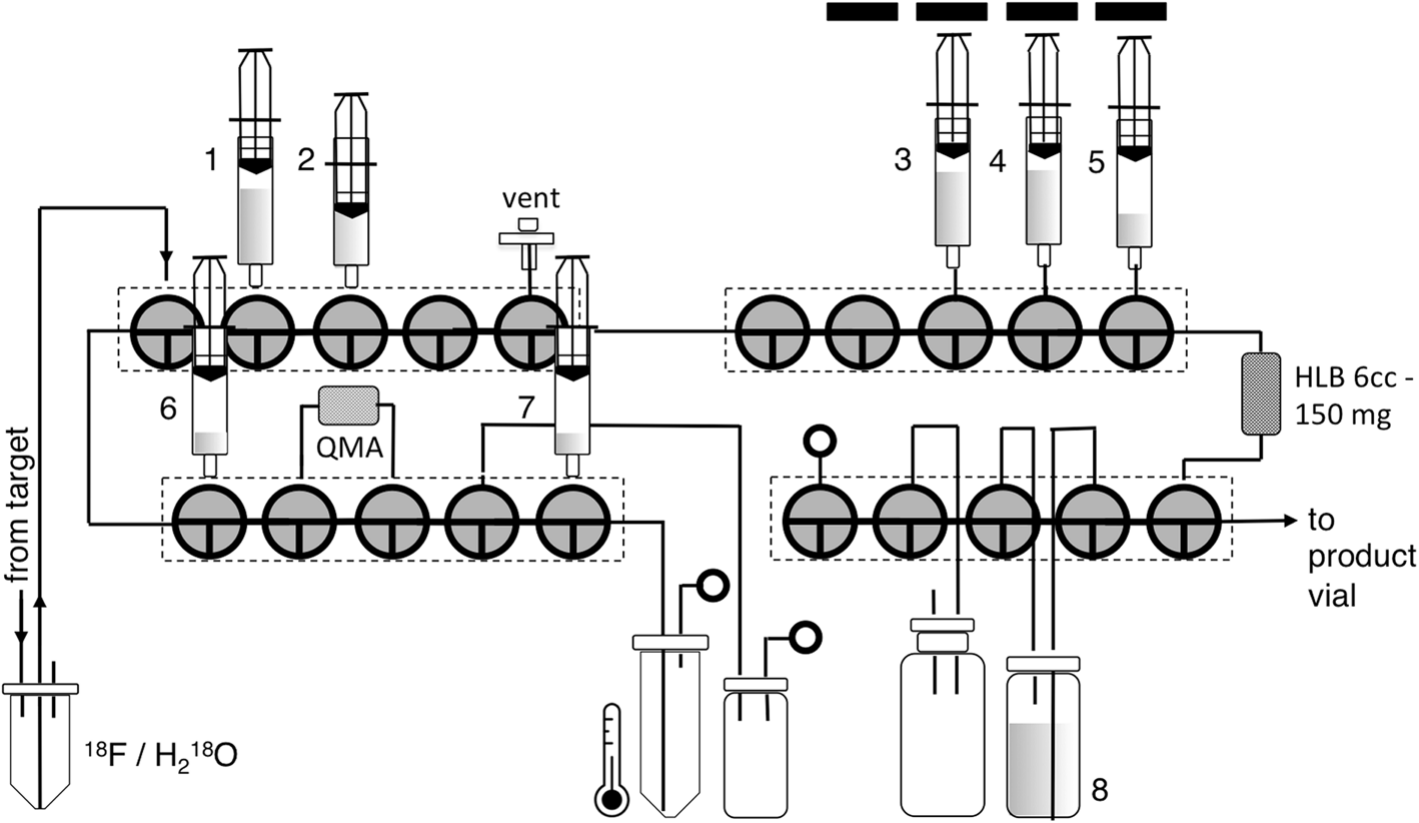
PiBOX cassette setup for production of [^18^F]AlF-P16-093. 1. 10 mL ultrapure water; 2. 6 mL water; 3. 10 mL water; 4. 10 mL saline; 5. 3 mL 2:1 ethanol: phosphate buffer; 6. 0.7 mL 4:1 pH 4.5 0.5 M NaOAc buffer: ethanol; 7. P16-093 in 0.45 mL ethanol; 8. formulation vial with 15.8 mL phophate buffer and 1.2 mL 14% saline. The black rectangles above syringes 3–5 represent linear actuators used for flow controlled SPE rinsing and elution

The automated radiosynthesis system (PiBOX) is a cassette-based, 1-pot device with solid phase extraction (SPE) workup capability (see Fig. 2). A typical configuration includes 20 actuators controlling 4 single-use, commercially available cassettes, each made of 5 ganged stopcocks. It was designed specifically for 1-pot reactions that do not require HPLC purification (e.g. Yao et al. 2019; Zhao et al. 2019), and supports standard ^18^F-recovery and drying methods typical of ^18^F-displacement reactions. A design goal was more consistent removal of chemical impurities using flow controlled SPE workup. PiBOX includes 4 linear actuators for flow-controlled rinsing and recovery of the product from an SPE cartridge using standard sterile, disposable 5–20 mL syringes. PiBOX is based on the Raspberry Pi Zero 2 W microcomputer (https://www.raspberrypi.com) and uses three commercially available Raspberry Pi HATs (hardware attached on top) to interface the servomotors for stopcock control and the reactor’s heater and thermocouple. Configuration, control and sequence generation software was programmed in the Python3 programming language (https://www.python.org/download/releases/3.0/). Figure 2 shows the fluidic hardware configuration of PiBOX for this work. Each synthesis utilizes a disposable set of 4 stopcock manifolds with associated tubing and fittings, glass vessels, sterile syringes and SPE cartridges.

### PiBOX yield optimization using ^18^F-rinses

Experiments were carried out using three different newly constructed PiBOX machines to determine the effects of important reaction parameters including reagent amounts, time, temperature and reagent sequencing on the overall radiochemical yield in the following manner. 2 mL of ultrapure water was introduced into a 5 mL conical glass vial that was rinsed previously with 1–2 mL ultrapure water before every experiment. The entire ^18^F-rinse volume (~ 0.5–1 mL; ~ 0.2–2 GBq) was transferred by hand using a metal needle and syringe and added to the conical vial. ^18^F-activity in the vial was assayed using an ion chamber before execution of the automated radiosynthesis. The following steps were then carried out automatically. The ^18^F-activity was transferred by vacuum through a Sep-Pak© Light QMA or QMA carb cartridge that had been conditioned with 10 mL of pH 4.5 0.5 M NaOAc buffer followed by 10 mL ultrapure water. The cartridge was rinsed with 10 mL of trace metal grade water then purged with air for 30 s by vacuum. The QMA cartridge was rinsed with 0.7 mL of 20–33% ethanol in pH 4.5 0.5 M NaOAc buffer to elute the trapped 18F activity into the reaction vessel by applying vacuum. Previously, the reaction vessel was loaded with AlCl_3_·6H_2_O (20–160 nmoles; 2–16 µL; 10 mM AlCl_3_·6H_2_O). In some experiments the AlCl_3_·6H_2_O was freeze-dried inside the bottom of the reaction vessel by aliquoting 2–16 µL AlCl_3_·6H_2_O (10 mM) into a reaction vessel and placing it into the freeze drier. After freeze drying, the AlCl_3_·6H_2_O-containing reaction vessels were stored at room temperature until use. After elution of the ^18^F-activity from the QMA into reaction vessel containing either the freeze-dried or liquid AlCl_3_·6H_2_O, the solution was heated for 5 min @ 50–65 °C then cooled for 30 s using compressed gas to ~ 35–45 °C. P16-093 ligand (80–160 nmoles) in 0.45 mL ethanol was added to the cooled solution and heated for 2–5 min @ 35–65 °C. In some experiments P16-093 was dissolved in 0.35 mL ethanol and 0.1 mL pH 4.5 0.5 M NaOAc buffer. In all cases the final P16-093 solution was ~ 1:1 pH 4.5 0.5 M NaOAc buffer:ethanol when combined with the QMA eluent. After heating the reaction mixture was cooled for 60 s to ~ 25–30 °C. This sequence of reagent addition and heating was named “two step” as [^18^F]AlF^2+^ is presumably formed first (Step 1) before reaction with P16-093 (Step 2).

For a subset of experiments radiolabeling was carried out using a “one-step” method where the ^18^F-activity eluted from the QMA and the AlCl_3_·6H_2_O were not heated separately from the P16-093. Instead, P16-093 in 0.45 mL ethanol was added to the reaction vessel immediately after elution of the QMA so that the [^18^F]F^−^, AlCl_3_·6H_2_O and P16-093 were all heated together for 5–10 min @ 40–50 °C. After heating the mixture was cooled for 60 s using compressed gas to ~ 25–30 °C.

After labeling (either 1 or 2 step) the reaction mixture was purified by SPE using a single HLB 6 cc 150 mg cartridge. The HLB cartridge was conditioned before use with 10 mL ethanol followed by 10 mL water. The reaction mixture was first diluted with 6 mL of water then transferred using pressure onto and through the HLB cartridge. The cartridge was rinsed in the manner suggested by Kersemans et al. (2018), first with 10 mL saline then 10 mL water using 2 separate linear actuator driven syringes @ ~ 10 mL/min. The product was eluted form the HLB with 3 mL of 2:1 ethanol 5 mM phosphate buffer (pH 7–7.5) into a vial containing 15.8 mL 5 mM phosphate buffer (pH ~ 7) with 1.2 mL concentrated (14%) saline to adjust osmolality. The final solution, 20 mL, is sterilized by passing it through a Millex GV filter using pressure, though for optimization and scale up reactions in this work no filter was used and the solution was transferred directly into a product vial.

At the end of an automated synthesis, the following components were assayed for ^18^F-activity using a dose calibrator: product vial (P), HLB cartridge (HLB), HLB waste (combined rinse and diluted reaction mixture volumes—HLB_w_), formulation vial (FV), QMA cartridge (QMA), QMA waste (combined rinse and ^18^F-conical vial volumes—QMA_w_), reaction vessel (R_x_V) and the ^18^F-conical vial (CV_r_).

For comparisons between runs a yield (RCY) was calculated using Eq. 1 below. All measurements were decay corrected to the time of measurement of the initial ^18^F-activity in the conical vial (CV_i_) at the start of synthesis.

1 $$RCY\left(\%\right)=\frac{P}{{CV}_{i}-{CV}_{r}}\times 100$$

A yield based on the activity eluted from the QMA was calculated using Eq. 2 to remove any effects of changing efficiency of ^18^F-recovery from QMA on the RCY. All activities were decay corrected to the time of measurement of starting [^18^F]fluoride in the conical vial.

2 $${RCY}_{QMA}=\frac{P}{{CV}_{i}-{CV}_{r}-QMA-{QMA}_{w}} \times 100$$

Finally, all the decay corrected component activities were summed, normalized to the starting ^18^F-activity and then expressed as a percent to determine the ^18^F-activity recovery (AR).

For large scale production ([^18^F]F^−^ > 30 GBq) the initial activity (CV_i_) was not measured so the RCY was calculated using the average AR from the ^18^F-rinse runs and the following formulas:

3 $$ALL=P+HLB+{HLB}_{w}+FV+QMA+{QMA}_{w}+{R}_{x}V+{CV}_{r}$$

4 $$RCY.LS (\%)=\frac{P}{(\frac{ALL}{AR}-{CV}_{r})} \times 100$$

5 $$RCY.{LS}_{QMA}(\%)=\frac{P}{(\frac{ALL}{AR}-{CV}_{r}-QMA-{QMA}_{w})}\times 100$$

The efficiency of QMA extraction was calculated by using the formula below for all combinations of QMA elution solution and QMA type:

6 $${QMA}_{eff}(\%)=\frac{{CV}_{i}-{CV}_{r}-QMA-{QMA}_{w}}{{CV}_{i}-{CV}_{r}}\times 100$$

### Time dependence of ^18^F-incorporation

To investigate directly if there was a time dependence (2–10 min) of ^18^F-incorporation into P16-093 under representative conditions of the PiBOX “two step” yield optimization studies the following experiment was carried out. Three PiBOX reaction vessels were prepared each containing 0.5 mL of 20% ethanol: pH 4.5 0.5 M NaOAc and 100 μg (80 nmoles) of P16-093 in 0.45 mL ethanol. [^18^F]AlF^2+^ was prepared automatically with PiBOX as described above using a reaction vessel with 120 nmoles of AlCl_3_·6H_2_O. A QMA carb cartridge was used to purify ^18^F-activity and 0.7 mL of 20% ethanol: pH 4.5 0.5 M NaOAc was used to elute the QMA cartridge. The mixture of buffer and AlCl_3_·6H_2_O was heated for 5 min @ 65 °C then cooled for 30 s to ~ 40 °C. The PiBOX sequence ended, and the reaction vial containing ~ 2 GBq [^18^F]fluoride was manually split into three equal aliquots (~ 200 μL). Each aliquot was added to one of the 3 previously prepared reaction vessels which were then placed in a preheated oil bath (40 °C) and sampled at 2, 5 and 10 min. Previously it was verified that the internal liquid temperature during PiBOX heating reached ~ 40 °C in 2–3 min. The samples were diluted with 10% ethanol with phosphate buffer (5 mM pH ~ 7) and put on ice until analyzed by both TLC and HPLC. The percent of total ^18^F-activity in the sample present as [^18^F]AlF-P16-093 was determined.

### Large scale production of [^18^F]AlF-P16-093

Radiosynthesis of [^18^F]AlF-P16-093 was carried out on a PiBOX machine (PiBOX-1) installed in a hot cell at the cyclotron facility at the University of Pennsylvania using the same program sequence and the same lots for reagents as in the companion low activity ^18^F-rinse runs. Three runs where carried out for each of two different “optimized” (reagents, time, temperature) conditions and the RCY’s and RCY_qma_’s from the low (^18^F-rinse) and high activity (~ 37 GBq [^18^F]fluoride @ cyclotron) production were compared to see if there was an effect of scaling up activity on radiochemical yield using a Student’s T-test. Tests using more than 37 GBq [^18^F]fluoride were not carried out. 37 GBq was considered an appropriate level to support on-site clinical investigations while minimizing exposure for this work, as all synthesis components were assayed for ^18^F-activity the same day for radiochemical yield and other efficiency estimates.

### Stability of final product

The stability of the final product solution was determined by sampling the product vial for up to 5 h after end of synthesis and analyzing the ^18^F-activity by thin layer chromatography (TLC) or HPLC. Stability was determined for storage at room temperature (RT) and 4 °C for representative low-level ^18^F-syntheses starting with 160 nmoles P16-093, and for 3 large scale production runs using 120 nmoles (RT only).

### Storage of reagents

P16-093 starting material was stored as either individual aliquots (100–200 µg; 80–160 nmoles) for each run at − 20 °C, or as an ethanol solution (2 mg/mL) stored at − 20 °C. Aliquots were prepared by making a 1 mg/mL solution of P16-093 in ethanol then removing the solvent from 100 to 200 µL aliquots. AlCl_3_·6H_2_O (10 mM) stock solution was stored at 4 °C. For experiments using freeze-dried AlCl_3_·6H_2_O, aliquots (2–16 µL) were added directly to the PiBOX reaction vessel and the solvent removed using a freeze drier. The freeze-dried vessels were stored at RT.

## Results

### PiBOX yield optimization studies using ^18^F-rinses

Based on published conditions for large scale production of [^18^F]AlF-PSMA-11 (Kersemans et al. 2018) and experience with the manual preparation of [^18^F]AlF-P16-093 (Zha et al. 2021), initial conditions for labeling were set at 80 nmoles AlCl_3_·6H_2_O with ~ 160 nmoles P16-093 (200 µg) using 65 °C for 5 min for both [^18^F]AlF^2+^ formation and P16-093 labeling carried out in two separate steps. Table 1 shows the results from a series of experiments carried out from October 2022 through July 2023 using 3 different PiBOX machines under varying reaction conditions and reagent preparations. Table 1 suggests a trend towards higher yields using freeze dried AlCl_3_·6H_2_O with more mild (lower temperature and shorter time) P16-093 labeling conditions. For example, the average RCY_qma_ using liquid AlCl_3_ for runs 1–8 excluding run 6 (56 ± 1.9%) was statistically different (p = 0.00004) than the average RCY_qma_ of freeze-dried AlCl_3_ runs 9–11 (66 ± 1.6%). The maximum yield was obtained using 30 – 40 °C for labeling P16-093 for 2–5 min using the two-step method. A similar high yield (79 ± 1.5%) was observed using a one-step method (40–50 °C for 10 min; runs 21, 23 and 25), which was not statistically different (p = 0.54) when compared to the average yield for runs 17–19 (78 ± 2.1%).

**Table 1:** Results from “^18^F-rinse” optimization studies using 80 nmoles AlCl_3_·6H_2_O with ~ 160 nmoles P16-093

Run	PiBOX	[^18^F]AlF^2+^°C/min	Label °C/min	Starting F18 (MBq)	Product F18 (MBq)	RCY %	RCY_qma_ %	Radiochemical purity %	Reagent LOT* AlCl_3_/P16-093
1	1	65/5	65/5	250	100	49	54	97	LQ1/FL1
2	1	65/5	65/5	907	396	54	59	97	LQ1/FL1
3	1	65/5	65/5	873	366	51	57	97	LQ1/FL1
4	1	65/5	65/5	855	355	51	55	97	LQ1/FL1
5	1	65/5	65/5	377	155	51	55	96	LQ1/FL1
6	1	65/5	65/10	659	189	36	38	97	LQ1/FL1
7	1	65/5	65/5	1406	595	52	58	98	LQ1/FL1
8	2	65/5	65/5	115	48	51	55	97	LQ1/FL2
9	2	65/5	65/5	1177	581	61	65	98	FD1/FL2
10	2	65/5	65/5	414	196	58	67	97	FD1/FL2
11	2	65/5	65/5	866	422	60	68	98	FD1/FL2
12	2	65/5	65/5	1302	696	66	72	98	FD1/FA3
13	2	65/5	65/5	295	121	50	54	97	FD1/FA3
14	2	65/5	65/5	929	455	60	66	97	FD1/FA3
15	2	65/5	65/2	995	566	68	76	96	FD1/FA3
16	2	65/5	50/2	648	340	64	67	96	FD1/FA3
17	2	65/5	40/2	733	448	74	79	96	FD1/FA3
18	2	65/5	40/2	770	474	74	80	96	FD2/FA3
19	2	65/5	40/2	703	411	71	76	96	FD2/FA3
20	2	65/5	30/2	733	407	67	72	97	FD2/FA3
21	2	–	40/10	1443	844	72	78	96	FD2/FA3
22	2	–	40/5	1632	821	60	66	97	FD2/FA3
23	2	–	50/10	1258	762	74	81	97	FD2/FA3
24	2	–	50/5	268	152	67	73	96	FD2/FA3
25	2	–	50/10	1258	762	74	79	97	FD2/FA3
26	2	50/5	50/5	1628	770	58	65	97	FD2/FA3
27	2	65/5	35/5	1206	692	71	79	96	FD2/FA3
28	3	65/5	40/2	1443	337	28	30	94	FD2/FA3
29	3	65/5	40/2	1591	836	64	67	96	FD3/FA3
30	3	65/5	40/2	2172	1099	61	65	97	FD3/FA3
31	3	65/5	40/4	477	244	62	66	97	FD3/FA4
32	3	65/5	40/4	429	244	70	75	97	FD3/FA4
33	3	65/5	40/4	755	422	69	74	97	FD3/FA4

*LQ#, liquid AlCl_3_·6H_2_O batch #; FD#, freeze-dried AlCl_3_·6H_2_O batch #; FL#, P16-093 stored in solution (2 mg/mL) in freezer batch #; FA#, P16-093 stored as solid (100–200 µg) in freezer batch #. Each batch # is a new series of aliquots from the same stock solution of (AlCl_3_·6H_2_O) or the same lot of P16-093 bulk material

Based on the preliminary optimization results in Table 1, a standard method for all PiBOX devices was chosen for further optimization studies. The standardized procedure consisted of a two-step synthesis using 65 °C for 5 min for [^18^F]AlF^2+^ formation followed by labeling of P16-093 for 4 min at 40 °C. Freeze-dried AlCl_3_·6H_2_O and P16-093 aliquots (100–200 µg) that were stored as a solid at -20 °C were used for the standardized method, and 1:4 ethanol/0.5 M NaOAc (pH 4.5) was used to elute activity from the QMA cartridge.

**Fig. 3:**
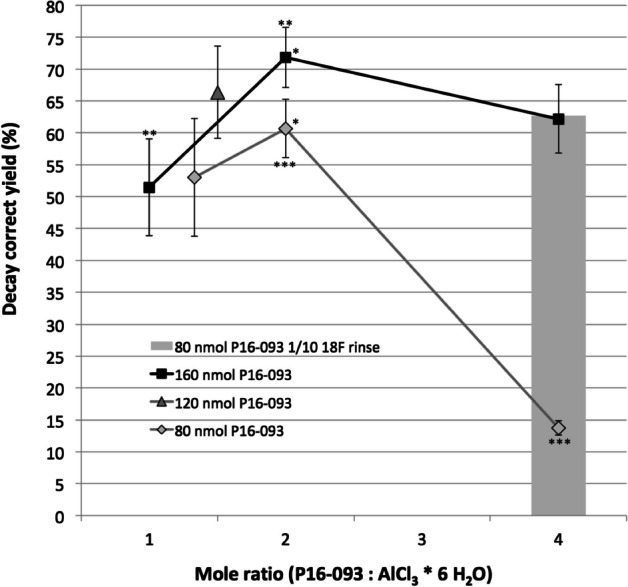
Effects of starting P16-093 and mole ratio of P16-093: AlCl_3_·6H_2_O on yield using optimized PiBOX reaction conditions. N = 3–4 for each data point. *P < 0.05; **P < 0.02; ***P < 0.0001. The bar graph represents results from a single experiment using the same conditions and reagents as the 4:1 mol ratio (80 nmol P16-093) runs except that the ^18^F-rinse volume was diluted with ultrapure water and sampled so that ~ 1/10 of the original ^18^F-rinse activity and volume was used for PiBOX

Figure 3 summarizes the results from experiments designed to determine the sensitivity of PiBOX RCY_qma_ to changes in starting P16-093 amount and relative amount of AlCl_3_·6H_2_O using the standardized conditions. There was a small but statistically significant decrease in yield for the 2:1 mol ratio of P16-093: AlCl_3_·6H_2_O when comparing 200 µg to 100 µg (P < 0.05), and also a trend towards lower yields when the mole ratio of P16-093: AlCl_3_·6H_2_O was decreased from 2:1 to ~ 1:1 using the same P16-093 mass. Using 200 µg P16-093, the decrease in yield from 2:1 to 1:1 was statistically significant (P < 0.02). Finally, at higher mole ratios (4:1), data from Fig. 3 suggests a trend (P = 0.06) towards slightly lower yields using 200 µg (160 nmol), but a significant (P < 0.0001) drop off for 100 µg (80 nmol) where the yield using a ratio 4:1 was only 13.7 ± 1.1% compared to 61 ± 4.6% using a mole ratio of 2:1. This large decrease in yield was not observed when only ~ 1/10 of the starting ^18^F-rinse activity (~ 55 MBq) was used with the exact same conditions and reagents (80 nmoles P16-093; 20 nmoles AlCl_3_·6H_2_O) where the yield was 62.4% in a single experiment (bar graph in Fig. 3).

### Time dependence of P16-093 labeling after [^18^F]AlF^2+^ formation

**Fig. 4:**
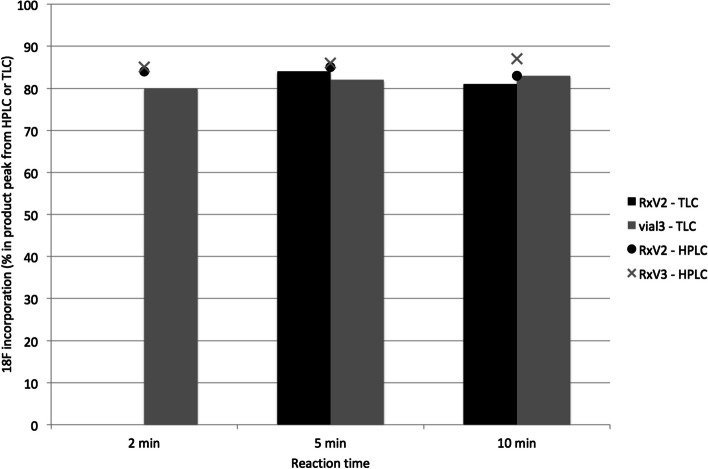
Time dependence of ^18^F-incorporation after incubation (40 °C) of 100 µg P16-093 (0.45 mL ethanol + 0.5 mL 20% ethanol in pH 4.5 0.5 M NaOAc buffer) with an aliquot (~ 1/3) of a reaction vessel that was reacted with QMA-purified [^18^F]fluoride and 120 nmoles AlCl_3_·6H_2_O using PiBOX. The final ratio of P16-093:AlCl_3_·6H_2_O was ~ 2:1 in each of 3 vials used for the time dependence study

The effect incubation time of [^18^F]AlF^2+^ with P16-093 on ^18^F-incorporation @ 40 °C is illustrated in Fig. 4. There was no difference in the percent of ^18^F-activity that was present as [^18^F]AlF-P16-093 from 2 to 10 min for two independent reactions measured by either HPLC or TLC. Only 2 of the 3 separate reaction vials prepared were used due to technical problems (1 vial lost to oil bath).

### Large scale production of [^18^F]AlF-P16-093

Table 2 shows the results of the large scale (> 30 GBq [^18^F]F^−^) production runs using the optimized PiBOX conditions. There was no difference in the average RCY_qma_ of the large-scale runs compared with the companion (same lot for all reagents) low-level ^18^F-rinse runs. For example, for the mole ratio of 2:1 P16-093: AlCl_3_·6H_2_O with 200 µg P16-093, the ^18^F-rinse runs gave a yield of 72 ± 4.7% while the scaled up runs using the same reagents had an average yield of 72 ± 4.3% (P = 0.97). A similar result was obtained when comparing low-level fluoride-18 runs using a mole ratio of 1.5:1 (150 µg P16-093) with the associated high ^18^F-activity runs: 66 ± 7.3% versus 66 ± 6.0% respectively. Finally, there were no differences in starting activity or yield between the two sets of high activity runs (Table 2), but there was a significant (P < 0.05) increase in molar activity from 110 ± 14 GBq/µmole to 140 ± 11 GBq/µmole when less P16-093 was used.

**Table 2:** Results from scaled up production runs using optimized conditions

Run	P16-093 (nmol)	AlCl_3_·6H_2_O (nmol)	Starting F18 (GBq)	Product F18 (GBq)	EOS %	RCY.LS_qma_ %	Molar activity* (GBq/µmole)	Radio-Purity %
1	160	80	31.5	15	48	67	94	98
2	160	80	33.2	18.3	55	75	114	99
3	160	80	35.5	19.3	54	73	120	99
4	120	80	32	17	54	73	145	98
5	120	80	38	18	47	64	147	97
6	120	80	34	15	45	61	127	97

*Molar activity is calculated assuming 100% of the starting P16-093 mass is in the final product vial. Radiochemical purity was determined by TLC. EOS (End of Synthesis) is the not decay corrected yield

### Stability of [^18^F]AlF-P16-093 preparations

**Fig. 5:**
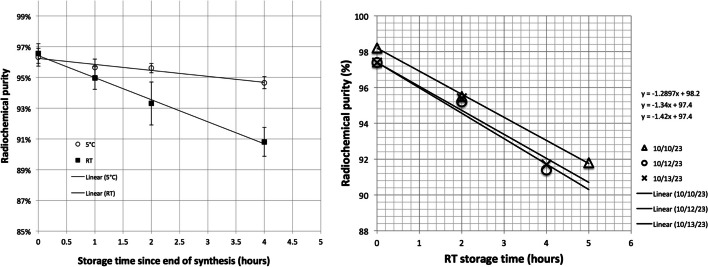
Left plot: summary of stability data of [^18^F]AlF-P16-093 stored at RT (square) and ~ 5 °C (circle) from low F18 activity optimization runs (N = 3–4) using 200 μg P16-093 and 80 nmoles AlCl_3_·6H_2_O. Right plot: individual run stability data from RT storage of [^18^F]AlF-P16-093 from 3 high activity (product > 15 GBq) production runs using 150 μg P16-093 and 80 nmoles AlCl_3_·6H_2_O. Product formulation is 10% ethanol in 5 mM phosphate buffer ~ pH 7

Radiochemical purity determined by TLC as a function of time is ploted in Fig. 5 for room temperature (RT) and refrigerator (~ 4 °C) storage conditions (left panel). The data was compiled from 5 different low level ^18^F-optimization runs on PiBOX using 200 μg of P16-093 and 80 nmoles AlCl_3_·6H_2_O. In two runs the product vial was split at the end of synthesis and stored at two different temperatures. In other experiments 100% of the product vial was stored at RT (N = 2) or in a refrigerator (N = 1). Data from each sample time point for all experiments was averaged and plotted together. Rates of loss of [^18^F]AlF-P16-093 were determined to be ~ 1.4%/h and ~ 0.4%/h for RT and refrigerator storage conditions respectively using the slopes of the linear trend lines shown in Fig. 5 left panel. The radiochemical purity as a function of time stored at RT is shown in Fig. 5 right panel for the 3 high activity runs 4–6 listed in Table 2. The average decrease in radiochemical purity was 1.34 ± 0.07%/h (average of the 3 individual trend line slopes).

### QMA and HLB extraction efficiencies

During 66 optimization runs 2 different QMA types were used with 2 different elution solutions. Table 3 summarizes the efficiencies for each case.

**Table 3:** Results from optimization runs using 3 different PiBOX machines

QMA type	Eluent	N	Efficiency	P-QMA*	P-Eluent*
QMA carb	33%	5	85.8 ± 1.7%		
QMA (Cl^−^)	33%	15	91.2 ± 2.0%	< 0.00005	
QMA carb	20%	19	90.5 ± 2.0%		< 0.001
QMA (Cl^−^)	20%	27	92.4 ± 1.6%	< 0.002	< 0.05

*A Student T-Test was used to compare efficiencies between QMA types for the same eluent composition (P-QMA), and between eluent compositions for the same QMA type (P-Eluent). Eluent percent is percent ethanol in a 0.7 mL mixture with pH 4.5 0.5 M NaOAc buffer

After QMA purification of [^18^F]F^−^ by QMA SPE, reaction of [^18^F]F^−^ with AlCl_3_·6H_2_O and ^18^F-labeling of P16-093, purification was carried out by flow-controlled SPE using an HLB 6 cc (150 mg) cartridge similar to the method described by Kersemans et al. (2018). The average ^18^F-activity (% of total starting activity) remaining on HLB was 1.7 ± 0.5% (N = 67) for the optimization runs. The waste from the HLB was assayed for [^18^F]AlF-P16-093 by TLC (0.6 ± 0.2%; N = 6). For the six runs where the HLB waste was assayed for [^18^F]AlF-P16-093, an HLB efficiency for recovery of [^18^F]AlF-P16-093 calculated to be 97.5 ± 1.1%, assuming all the activity remaining on the HLB was [^18^F]AlF-P16-093.

## Discussion

[^18^F]AlF-P16-093 seeks to leverage the simple and high yield method of [^18^F]AlF^2+^ (McBride et al. 2009) to convert [^68^Ga]Ga-P16-093 into an ^18^F-analog that is suitable for wider clinical use while maintaining the imaging advantages demonstrated by [^68^Ga]Ga-P16-093 (Wang et al. 2023). Though previously synthesized manually for pre-clinical evaluation (Zha et al. 2021), [[^18^F]AlF-P16-093 has not been systematically optimized on an automated platform and scaled up for “clinical-level” production (e.g., 37 GBq ^18^F]F^−^).

Scaling up and/or automating radiochemical reactions based on manual syntheses is not always straight forward (Kersemans et al. 2018; Wurzer, et al. 2021). In particular, manual reactions using [^18^F]AlF^2+^ for chelation labeling (AlF) or [^18^F]F^−^ for isotopic exchange (IE) reactions typically fractionate ^18^F-activity from the target such that only a small proportion of the target volume (and associated impurities like metal ions) is used. Combined with very small reagent volumes (2–20 uL), final reaction volumes in manual [^18^F]AlF^2+^ reactions can be very small (0.2–0.5 mL). These attributes are often not amendable to traditional automation and lower yields are not uncommon when scaling up with machines (Kersemans et al. 2018; Wurzer et al. 2021).

Manual reactions that fractionate [^18^F]F^−^ may also not represent the true levels of [^19^F]fluoride present. While 37 GBq of [^18^F]fluoride is only ~ 0.5 nmoles based on the radionuclide’s theoretic specific activity, practical molar activities of final products suggest that [^19^F]fluoride mass is 5–50 nmoles or more (Cleeren et al. 2016; Link et al. 2012), as [^19^F]fluoride is known to contaminate [^18^F]fluoride production from several sources including delivery lines and reagents (Link et al. 2012). This amount of [^19^F]fluoride can be significant proportion of reagent amounts in AlF and IE reactions where precursor amounts are often 100–200 nmoles. Cleeren et al. (2016) demonstrated this directly by titrating AlF reactions with [^19^F]fluoride under standard AlF labeling conditions (40 nmoles AlCl_3_; 150 nmoles ligand), showing a decrease in RCY when > 40 nmoles [^19^F]F^−^ is added. Wurzer et al. (2021) calculated that [^19^F]fluoride can be present at > 10 nmoles just from typical target water volumes based on known specifications, and therefore [^18^F]F^−^ molar activity may impact IE RCY directly where only 150 nmol precursor is used (Wurzer et al. 2021).

Accordingly, AlF optimization studies should be carried-out using the same production conditions as in clinical production  to reproduce impurity and [^19^F]fluoride concentrations that can affect RCY. This is not always practical and, in this work, 100% of “^18^F-rinse” volumes were used instead for optimization. ^18^F-rinses (H_2_^18^O) used about ½ the normal target volume and traversed the same radionuclide delivery path including delivery lines, target body and other standard plumbing shortly after a clinical production run. The RCY of large-scale runs were not different than ^18^F-rinse optimization runs using 80 nmoles of AlCl_3_. At lower AlCl_3_ levels (20 nmoles with 80 nmol P16-093), we observed a significant decrease in RCY that was not observed when an ^18^F-rinse was “fractionated” (Fig. 3 bar graph). Given that the RCY was high using 80 nmol P16-093 with 40 nmol AlCl_3_, we attributed the severe decrease in yield to [^19^F]F- and/or impurities such as metal ions present in the ^18^F-rinse that competed or otherwise interfered with AlCl_3_ when only 20 nmoles was used. We concluded that the minimum amount of AlCl_3_ for high yield under our large-scale production conditions was ~ 40–80 nmoles. Only 80 nmoles was used to validate large-scale production, though Fig. 3 results suggest that 40 nmoles may also work with only a small decline in RCY. Moreover, the effect of aliquoting [^18^F]fluoride (Fig. 3 bar graph) validated our approach to use 100% of an [^18^F]F-rinse as a better proxy for real production impurity/[^19^F]F-concentrations, though greatly limiting throughput. (1 rinse-run/day).

A precursor-to-AlCl_3_ mole ratio range of ~ 2:1 gave the optimal RCY and is similar to literature results for [^18^F]AlF-PSMA-11 (Kersemans et al. 2018). Slightly lower mole ratios also gave high yields and molar activity was improved significantly using 120 nmoles of P16-093 with 80 nmoles of AlCl_3_ (see Table 4). No difference in RCY or radiochemical purity was observed using a one-step versus a two-step reaction during optimization. Though some investigators report that a two-step reaction is necessary for high yields and/or high purity using [^18^F]AlF^2+^ with PSMA-11 (Kersemans et al. 2018), this result is consistent with other [^18^F]AlF-PSMA-11 reactions (Malik et al. 2015; Al-Momani et al. 2017).

**Table 4:** Large-scale [^18^F]AlF^2+^ labeling of acyclic chelators conjugated with an urea-based PSMA inhibitor

Machine Reference	Compound	AlCl_3_nmol	Ligand nmol	Product (GBq)	EOS yield (%)*	Molar activity (GBq/µmole)	Purification	Time (min)
SynthraFCHOL Kersemans (2018)	PSMA-11	100	200	24 ± 6	21 ± 3%	120 ± 28	SPE (HLB × 2) 360 mg	35
TracerLab FX Giglio (2018)	PSMA-11	45	60	2.7–17.4	18%	58–544	Semi-prep HPLC	Not reported
Custom remote Cleeren (2016)	Glu-NH-CO–NH-Lys(Ahx)L3	100	300	8.14	25%***	27	Semi-prep HPLC	35**
PiBOX	P16-093	80	160	17.5 ± 2.2	52 ± 4%	109 ± 14	SPE (HLB × 1) 150 mg	30
PiBOX	P16-093	80	120	16.7 ± 1.3	49 ± 5%	140 ± 11	SPE (HLB × 1) 150 mg	30

*End of Synthesis (not decay corrected)

**Does not include time to trap and recover [^18^F]F^−^ on QMA

***Not optimized

QMA (chloride form) was the preferred type (Table 3) compared to QMA (carb), though the difference was very small, particularly when the 20% ethanol eluent was used. In general, recovery of activity from the QMA using 0.7 mL of 20% ethanol in 0.5 M NaOAc in the optimization runs was consistent with published values that use 0.5–0.6 mL of 100% 0.5 M NaOAc (Kersemans et al. 2018; Giglio et al. 2018).

Some investigators report that purification of [^18^F]AlF-PSMA-11 by SPE was not possible due to excessive breakthrough during automated synthesis and HPLC was used instead (Giglio et al. 2018). Kersemans et al. (2018) also described significant (> 25%) breakthrough using many standard types of SPE bonded phases and proposed using 2 SPE cartridges (HLB 360 mg) in tandem to get > 98% retention. In this work, a single, larger format HLB (6 cc; 150 mg) was sufficient for nearly quantitative recovery using flow-controlled rinsing. Ethanol content of the reaction mixture after dilution before loading the SPE cartridge was ~ 8% and was similar to Kersemans et al. (2018).

The radiochemical purity of the final product remained > 90% when stored at RT for ~ 4 h and exhibited a rate of decline in radiochemical purity similar to Kersemans et al. (2018) for [^18^F]AlF-PSMA-11 using a similar formulation. Refrigeration slowed decomposition (loss of ^18^F-label) by about threefold to ~ 0.4%/h. The decline in radiochemical purity over several hours could limit patient throughput. This can be compensated in part by refrigeration, where 14 patients could be done a single scanner while maintaining purity >  = 95% (assuming starting purity is 98%) where patients are scanned every 30 min after 60 min is allowed for QC, packaging and delivery—all refrigerated. This number would reduce to 9 patients/scanner if the dose was at room temperature during QC, packaging and delivery, but otherwise kept refrigerated, and only 3 patients/scanner if the dose was never refrigerated. Therefore, [^18^F]AlF-P16-093 (like [^18^F]AlF-PSMA-11) is most suited for local cyclotron-based hospital use and would require refrigeration for limited distribution.

The results of the large-scale [^18^F]AlF-P16-093 syntheses using PiBOX are compared with high activity production of similar compounds (acyclic chelators conjugated with a PSMA inhibitor) in Table 4. The yield not decay corrected was ~ 2 × higher than for [^18^F]AlF-PSMA-11 (Kersemans et al. 2018; Giglio et al. 2018) and Glu-NH-CO-NH-Lys(Ahx)L3 (Cleeren et al. 2016) with similar execution time using similar or less starting material. Given that many conditions used here were based in part on published methods for [^18^F]AlF-PSMA-11, this result was unexpected. The only known differences in method with automated [^18^F]AlF-PSMA-11 was the use of ethanol/NaOAc mixture to elute the QMA and the use of freeze-dried AlCl_3_·6H_2_O. Although we observed a benefit of freeze-dried AlCl_3_·6H_2_O, it was not large enough to explain the twofold higher yield. In general, reaction times and temperatures were similar to those used for labeling with commercial machines, although [^18^F]AlF-PSMA-11 methods that used a 2-step method used RT for reaction of [^18^F]F^−^ with AlCl_3_ (Kersemans et al. 2018; Giglio et al. 2018) compared to 65 °C used here. Given that conversion of [^18^F]F^−^ to [^18^F]AlF^2+^ was reported to be > 80% by Kersemans et al. (2018), this also likely does not explain the difference in overall yield.

## Conclusion

The optimized production of [^18^F]AlF-P16-093 facilitates further clinical implementation and wider adaptation. Its simple, efficient (> 50% none-decay-corrected) labeling at > 15 GBq levels, and desirable performance characteristics in PCa patients makes [^18^F]AlF-P16-093 an attractive alternative to existing PSMA-targeted tracers. The PiBOX system, made mostly from off-the-shelf, commercially available hardware and opensource software, is a robust and affordable platform for automated [^18^F]AlF-P16-093 production, may also be suitable for other automated radiochemical syntheses requiring only SPE purification.

## Data Availability

Most data generated or analysed during this study are included in this published article. The complete datasets used and/or analysed during the current study are available from the corresponding author on reasonable request.

## Abbreviations

AlF: [^18^F]AlF^2+^ for chelation labeling
HPLC: High performance liquid chromatography
IE: [^18^F]F^−^ for isotopic exchange
PET: Positron emission tomography
PCa: Prostate cancer
PSMA: Prostate specific membrane antigen
RT: Room temperature
SPE: Solid phase extraction
TLC: Thin layer chromatography
